# Bearing Capacity and Mechanism of the H–V Geogrid-Reinforced Foundation

**DOI:** 10.3390/polym15122606

**Published:** 2023-06-08

**Authors:** Juan Hou, Sitong Liu, Boohyun Nam, Yanxia Ma

**Affiliations:** 1 School of Mechanics and Engineering Science, Shanghai University, Shanghai 200444, China; liusitong@shu.edu.cn; 2 School of Civil Engineering, Qinghai University, Xining 810016, China; 2001990020@qhu.edu.cn; 3 School of Engineering, University of Virginia, Charlottesville, VA 22904, USA; 4 Department of Civil Engineering, Kyung Hee University, 1732 Deogyeong-daero, Giheung-gu, Yongin-si 17104, Republic of Korea; boohyun.nam@khu.ac.kr

**Keywords:** horizontal–vertical geogrid, foundation, bearing capacity, reinforced mechanism, strip footing

## Abstract

A series of model tests were conducted to investigate the bearing capacity and reinforced mechanism of a horizontal–vertical (H–V) geogrid-reinforced foundation. The bearing capacities of the unreinforced foundation, the conventional geogrid, and the H–V geogrid-reinforced foundation were compared. The parameters, including the length of the H–V geogrid, the vertical geogrid height, the depth of the top layer, and the number of H–V geogrid layers, are discussed. Through experiments, it was found that the optimal length of H–V geogrid is around 4B, the optimal vertical geogrid height is approximately 0.6B, and the optimal depth of the top H–V geogrid layer is between 0.33B and 1B. The optimal number of H–V geogrid layers is 2. The result also indicates that the bearing capacity of H–V geogrid is almost 1.7 times greater than that of conventional geogrid. Additionally, the maximum top subsidence of H–V geogrid-reinforced foundation decreased by 13.63% compared to that of conventional geogrid-reinforced foundation. Under the same settlement, the bearing capacity ratio of two H–V geogrid-reinforced foundation layers is 75.28% higher than that of one layer. The results also demonstrate that the vertical elements of H–V geogrid interlock the sand from being displaced under the applied load and redistribute the surcharge over a wider area, thereby increasing the shear strength and improving the bearing capacity of an H–V geogrid-reinforced foundation.

## 1. Introduction

Geogrids are commonly used in landfill construction to reinforce and stabilize the soil and waste material. Several investigators have widely demonstrated the beneficial effects of using conventional geogrid to increase the bearing capacity of the foundation [1,2]. Extensive laboratory model tests were conducted to assess the improvement of the bearing capacity of conventional geogrid-reinforced foundations [3,4]. Mandal and Manjunath studied the reinforcement effect of bamboo as vertical reinforcement under a strip foundation and found that this type of reinforcement improved the bearing capacity and stiffness of sand subgrade [5]. Wang et al. studied the dynamic test of square foundations on unreinforced and reinforced foundations. It was found that the bearing capacity of the foundation on reinforced foundation was at least 12% higher than that of unreinforced foundation [6]. Phanikumar et al. carried out a series of laboratory plate load tests of geogrid-reinforced sand beds under fine, medium, and coarse sand beds. They found that conventional geogrid reinforcement improved the load–settlement response [7]. Roy and Deb studied the influence of the aspect ratio of rectangular foundation and the thickness of the sand layer on the bearing capacity, settlement characteristics, load diffusion angle, and the size of the geogrid layer through the model plate load tests [8]. Binquet and Lee [9] investigated the bearing capacity of a shallow foundation reinforced with metal strips under a strip footing. The results indicated that the bearing capacity could be improved by 200% compared to an unreinforced foundation. Guido et al. [10] reported that the optimum depth of the top layer is less than 0.75–1.0 times the footing width. Patra et al. [11] studied the behavior of strip footing constructed on reinforced clay. The settlement was found to be reduced with the increase in reinforcement size, stiffness, and the number of layers. Other parameters, including the location of the top layer, the depth of a reinforcement zone, the number of reinforcement layers, and the width of each reinforcement layer, were also investigated [12,13,14,15,16,17,18].

Considerable attention has been given recently to the development of three-dimensional reinforcement to increase the interface between the reinforcement and the soil [19,20,21,22,23,24,25,26]. Alamshahi and Hataf [27] investigated a new reinforcing element that attached grid anchors to conventional geogrid. They found that the grid anchors improve the bearing capacity scientifically. Mosallanezhad et al. [28] found that the ultimate bearing capacity of shallow square footing reinforced with grid anchors was increased by 3.0 times and 1.8 times, respectively, compared to unreinforced soil and soil reinforced with conventional geogrid. Makkar et al. [29] designed two different types of three-dimensional geogrids, namely triangular pattern and rectangular pattern, and studied the factors that affected the bearing capacity of the foundation, such as the depth of the top layer, the spacing between layers, and the number of layers. Zhang et al. and Hou et al. [30,31,32] proposed H–V strips with no open grid and found that H–V strips perform better than conventional planer ones. Hou et al. [33] indicated that vertical stripes provide passive resistance against soil, significantly increasing the shear strength of the soil. Zhang et al. [34] studied the influence of H–V strips on the bearing capacity of the foundation through a series of model tests. Hou et al. [35] further investigated the effect of the depth of single-layer strips and the number of strip layers on the bearing capacity of the H–V strip-reinforced foundation. 

Abu-Farsakh et al. [36] emphasized that the geometrical open characteristics of a geogrid were more critical than its tensile strength. Referring to this recognition, this study first develops a new 3D H–V geogrid and investigates its bearing capacity when used as a reinforcement foundation. The primary importance of H–V geogrid is that it is a kind of 3D reinforcement composed of horizontal and vertical geogrids with open-size geometry. The differences between the performance of the unreinforced, conventional geogrid-reinforced, and H–V geogrid-reinforced foundation were compared. The effects of the top layer, the length of the reinforcement, vertical geogrid height, and the number of H–V geogrid layers on the bearing capacity and the deformation of the foundation were discussed. The behavior of failure progressive and the possible mechanism of the H–V geogrid-reinforced foundation were explored.

## 2. Materials and Methods

### 2.1. Test Setup

**Figure 1:**
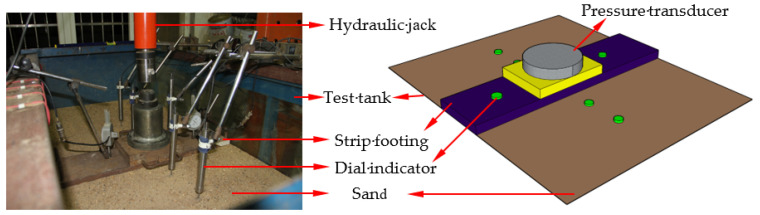
Picture and schematic diagram of the test setup.

Figure 1 shows the photo and the schematic diagram of the test setup. The inner dimensions of the test tank are 0.6 m (width) × 1.4 m (length) × 1.1 m (depth). The front and rear walls of the tank were composed of thick, double, see-through, smooth Perspex sheets. The left and right sides comprised a 16 mm thick, transparent Perspex sheet and 5 mm thick steel plate, respectively. A rigid steel plate (598 mm length, 150 mm width, and 25 mm height) was used as the strip footing. Each 0.01 m gap is left to prevent the footing from contacting the two inner surfaces of the test tank. The two ends of the footing plate were also polished to a smooth surface to minimize the effects of end friction. The footing width (B) is 0.15 m, about 10% of the tank length (L = 1.4 m), to simulate plane strain conditions. Two other steel plates with dimensions of 0.2 m × 0.15 m were laid on the strip footing (see Figure 1). The hydraulic jack with a 100 kN capacity vertically transferred the pressure to the footing. The data-acquisition system (DH3815) was used to synchronize the load and settlement. Two dial indicators with an accuracy of 0.01% of the full range (0.75 m) were placed on the footing model to measure the footing settlement during the loading process. Four dial indicators with an accuracy of 0.01% of the full range (0.35 m) were placed symmetrically along the length of the tank on the foundation surface to measure the surface displacement.

### 2.2. Test Materials

**Figure 2:**
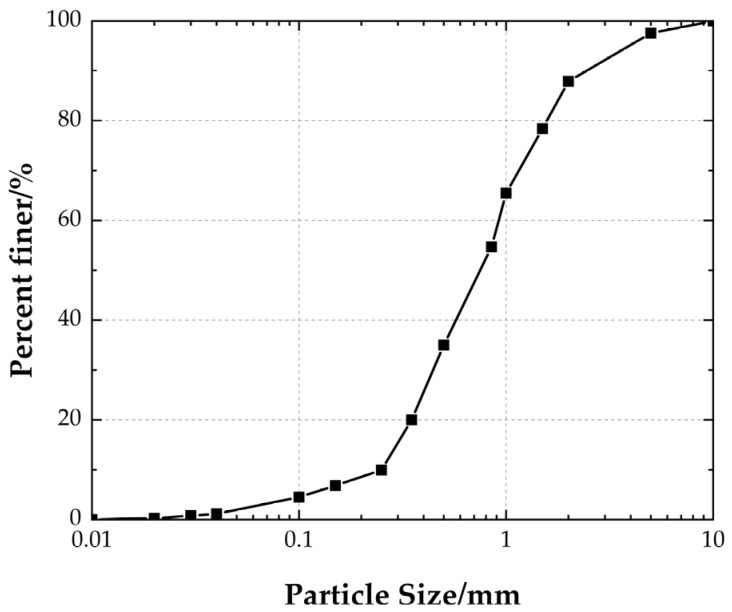
Gradation curve of sand.

The dry Huangpu River sand (Shanghai, China) was used throughout the investigation. Table 1 summarizes the physical properties of the sand. Figure 2 shows the particle size distribution curve for the sand. The uniformity coefficient (Cu) and coefficient of curvature (Cc) are 1.41 and 1.01, respectively. The measured angle of internal friction resistance is 35.2 degrees (direct shear tests).

**Figure 3:**
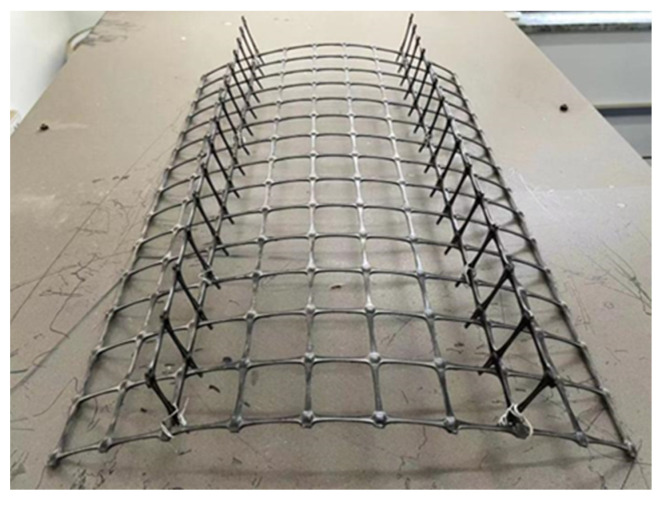
Example of H–V geogrid.

An H–V geogrid was made by fixing two vertical elements to the horizontal element every 0.225 m, as shown in Figure 3. All horizontal and vertical elements of the H–V geogrid were made using conventional geogrid. The geogrid is 2 mm thick. The grid size is 45 mm × 40 mm. The mechanical properties of the geogrid are given in Table 2.

### 2.3. Test Procedure

**Figure 4:**
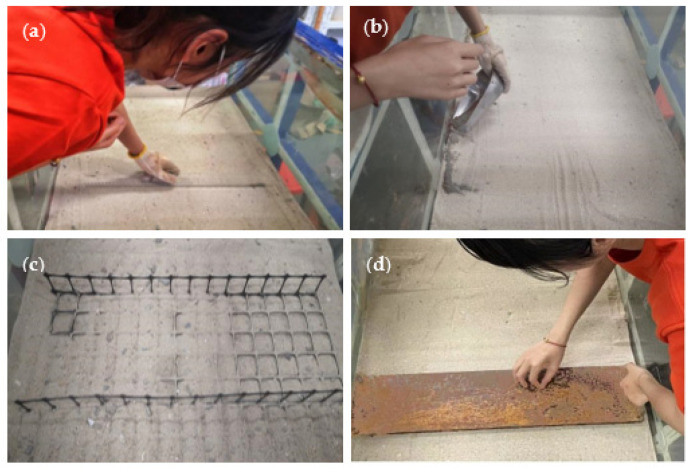
Experimental process: (**a**) Scrape the surface of the sand flat; (**b**) Mark ash lines; (**c**) Place the H–V geogrid; (**d**) Place the strip footing.

The dry sand was placed into the test tank and compacted into layers 0.05 m thick every time. The required amount of sand was weighed and placed into the test tank using a metal scoop to ensure the data quality. The surface of the sand was scraped flat (Figure 4a), and the sand was compacted to the specified position to achieve the designed density. Coal ash was placed on the tank’s inner surface to mark several horizontal and vertical dark lines to observe the critical slip surface of the sand (Figure 4b). The conventional or H–V geogrid was placed at the designed depth (Figure 4c). The procedure continued until the total desired height of sand was achieved. The sand surface was then leveled and the footing was placed (Figure 4d). Finally, the data acquisition and the static load systems were established, as shown in Figure 2. 

**Figure 5:**
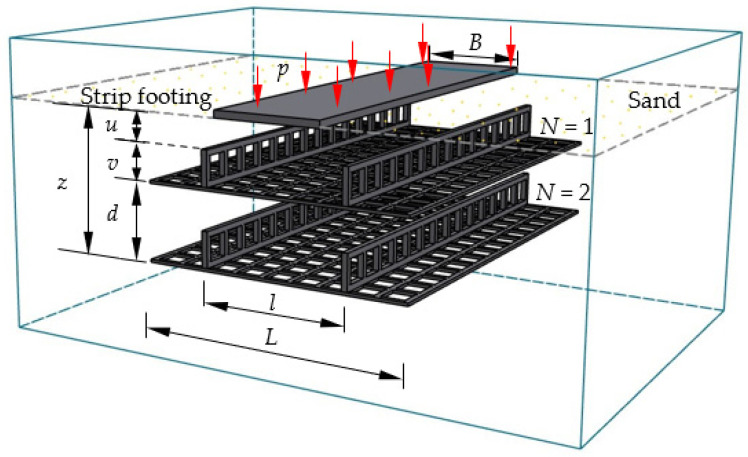
The typical layout of the multilayer H–V geogrid-reinforced foundation.

Figure 5 shows the typical layout of the multilayer H–V geogrid-reinforced foundation adopted in the model tests. *N* (the number of H–V geogrid layers) layers of reinforcement were placed at specific depths. The depth of the top layer is represented as *u*, the total depth of the H–V geogrid layers is *z*, the length of reinforcement is *L*, the distance between the two vertical elements is *l*, the vertical spacing of the layer is *d*, and the vertical geogrid height is *v*. To discuss this conveniently, *u*, *z*, *L*, *l*, *d,* and *v* were expressed as the time of the width of the strip footing (B). 

The test cases are designed into three groups, as shown in Table 3. A, B, and C represent the unreinforced, the conventional geogrid, and the H–V geogrid-reinforced foundation, respectively. For the unreinforced foundation, the comparison shows less than 8% agreement with Terzaghi’s bearing capacity theory [37]. All the performances of the cases were investigated in the range of footing settlement less than 0.2B (*s* = 30 mm) if there were no significant failures in the tests.

## 3. Results and Discussion

Performance improvement due to the geogrid can be represented using two nondimensional improvement factors: the bearing capacity ratio (*BCR*) and the settlement reduction factor (*SRF*). *BCR* represents the improvement in the bearing capacity of the foundation, which compares the bearing capacity of the reinforced foundation to that of the unreinforced foundation at a given settlement. *SRF* represents the reduction in settlement of the foundation, which compares the settlement of the reinforced foundation to that of the unreinforced foundation at a given bearing capacity.

The *BCR* and the *SRF* can be expressed as follows:

(1) $$BCR=\frac{p_{\mathrm{rein}}}{p_{\mathrm{un}}}$$

where *p*_rein_ is defined as the footing pressure of the reinforced soil and *p*_un_ is the footing pressure of the unreinforced soil.

(2) $$SRF=\frac{s_{\mathrm{rein}}}{s_{\mathrm{un}}}$$

where *s*_rein_ is the settlement of the reinforced soil and *s*_un_ is the settlement of the unreinforced soil.

### 3.1. Comparison of the Different Reinforced Foundation

**Figure 6:**
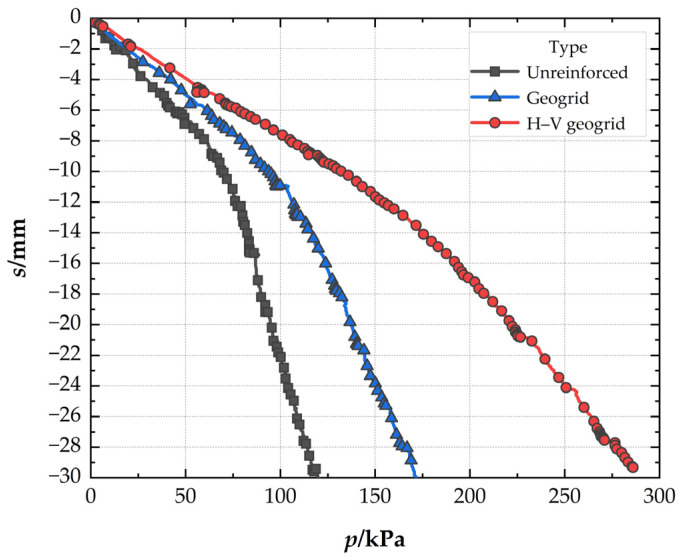
Pressure–settlement curves of the different foundations.

Figure 6 presents the pressure–settlement (*P*–*s*) curves of different foundations. It can be seen that, at the settlement of 0.03B (*s* = 4.5 mm), the bearing capacity is lower in unreinforced foundations (32.9 kPa) than that of conventional geogrid-reinforced foundations (48.02 kPa), and the *BCR* is 1.46 for the conventional geogrid-reinforced foundation. At the same settlement, the *BCR* is 1.78 for the H–V geogrid-reinforced foundation, which is 21.92% higher than the conventional geogrid-reinforced foundation. The bearing capacity, improved by the H–V geogrid (25.79 kPa), is almost 1.7 times greater than the conventional geogrid (15.12 kPa). However, the number of consumables of the H–V geogrid is only 1.2 times that of the conventional geogrid, which indicates that the H–V geogrid is more economical than the conventional geogrid. When the pressure is 119 kPa, the *SRF* is 49.5% for the conventional geogrid-reinforced foundation, whereas *SRF* is 29.6% for the H–V geogrid-reinforced foundation. Therefore, for the cases with the same arrangement, the H–V geogrid-reinforced foundation exhibits a higher stiffness (i.e., a greater slope of the *P*–*s* curve), a more significant bearing capacity, and a smaller settlement than the conventional geogrid-reinforced foundation.

**Figure 7:**
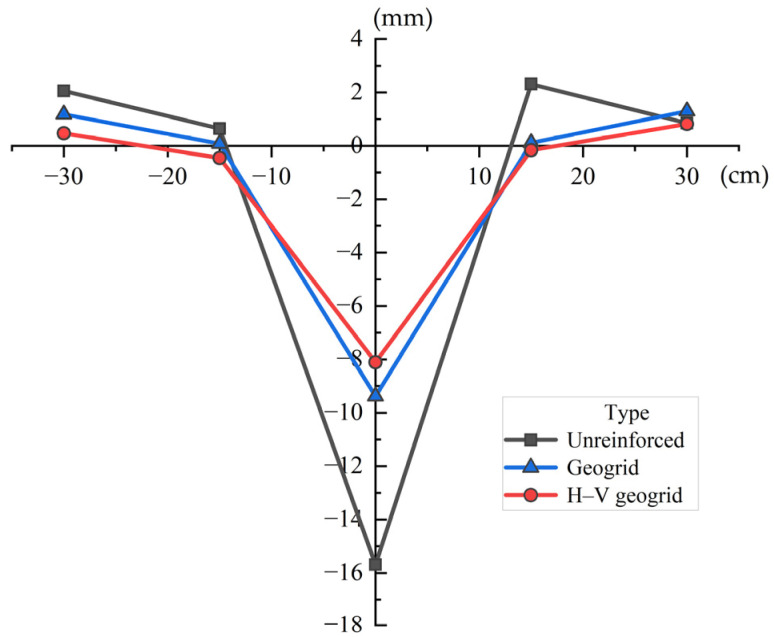
Surface deformation of the different foundations.

Figure 7 illustrates the relationship between the distance from the footing center and the deformation of the soil surface. The maximum surface uplift of the unreinforced foundation is 2.31 mm and the maximum surface subsidence is 15.69 mm. Meanwhile, the maximum surface uplift of the conventional geogrid-reinforced foundation is 1.30 mm and the top surface subsidence is 9.39 mm. The top surface subsidence of conventional geogrid-reinforced foundation decreases by 40.15% compared to that of unreinforced foundations. It can be seen that the geogrid can effectively reduce the settlement of the foundation. In addition, the maximum surface uplift is 0.82 mm and the maximum surface subsidence is 8.11 mm. The maximum top subsidence of H–V geogrid-reinforced foundation decreased by 13.63% compared to that of conventional geogrid-reinforced foundation. It can be seen that the performance of the H–V geogrid in reducing foundation settlement is better than that of the conventional geogrid. The H–V geogrid inhibits the development of rupture planes in the soil bed, thereby inducing a better composite behavior, which reduces surface heaving. Meanwhile, the sand surface undergoes more significant heaving and settlement in the conventional geogrid-reinforced case than in the H–V reinforced case (see red and blue lines in Figure 7). 

**Figure 8:**
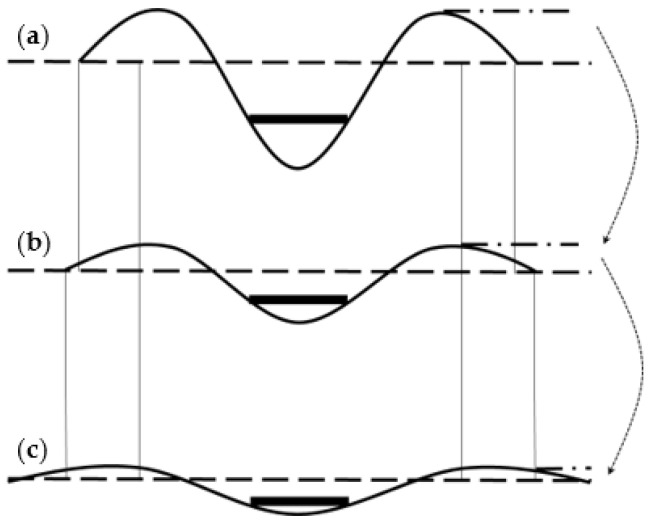
Schematic diagram of changes in the sand surface after the tests: (**a**) Unreinforced foundation; (**b**) Conventional geogrid-reinforced foundation; (**c**) H–V geogrid-reinforced foundation.

This trend can be further understood from the schematic diagram of the change in the sand surface deformation presented in Figure 8. From these comparisons, it can be concluded that the H–V geogrid greatly benefits the bearing capacity and settlement of the foundation, and the H–V geogrid-reinforced foundation behaves more uniformly. 

### 3.2. Effect of the Depth of the Top Layer

**Figure 9:**
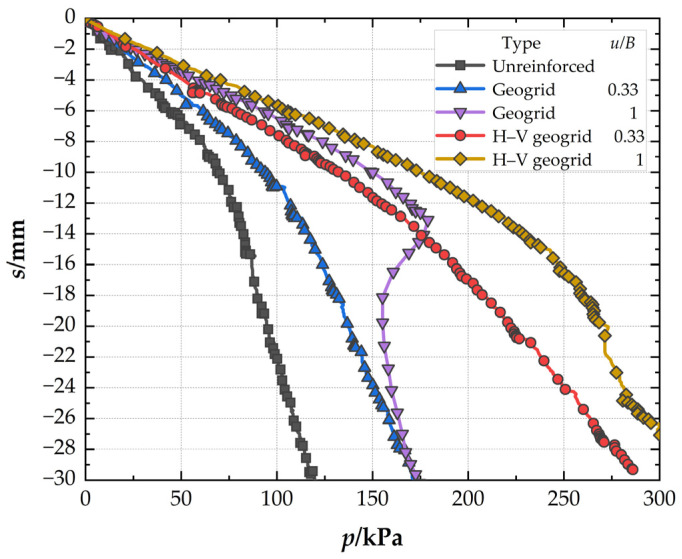
Pressure–settlement curves for the conventional geogrid and H–V geogrid with different depths of the top layer.

Figure 9 illustrates the effect of the depth of the top layer (*u*) on the *P*–*s* curves of conventional geogrid and H–V geogrid-reinforced foundations. At the settlement of 0.03B (*s* = 4.5 mm), when *u* is 0.33B, the *BCRs* of the conventional geogrid-reinforced foundation and H–V geogrid-reinforced foundation are 1.46 and 1.78, respectively. The *BCR* of the H–V geogrid-reinforced foundation is 21.92% higher than that of conventional geogrid-reinforced foundations. When *u* is 1B, the *BCRs* are 2.11 and 2.52 for the conventional geogrid-reinforced foundation and H–V geogrid-reinforced foundation, respectively. The *BCR* of the H–V geogrid-reinforced foundation is 19.43% higher than that of conventional geogrid-reinforced foundations. This shows that the bearing capacity improvement significantly depends on the depth of the top layer. At *p* = 119 kPa, when *u* is 0.33B, the *SRFs* of conventional geogrid-reinforced foundation and H–V geogrid-reinforced foundation are 29.6% and 49.5%, respectively. When *u* is 1B, the *SRF* is 25.87% for the conventional geogrid-reinforced foundation and 22% for the H–V geogrid-reinforced foundation. Therefore, at the same depth of the top layer (*u*), the performance of improving the bearing capacity of H–V geogrid is superior to that of conventional geogrid.

**Figure 10:**
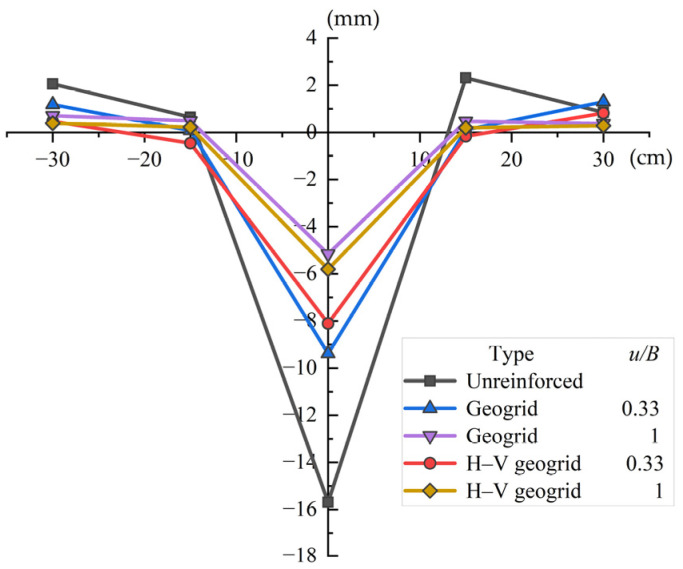
Surface deformation for the conventional geogrids and H–V geogrids with different depths of the top layer.

Figure 10 illustrates the surface deformations (heave/settlement) for the conventional and H–V geogrid-reinforced foundation with different depths of the top layer (*u*). When *u* is 0.33B, the maximum surface uplift of the H–V geogrid-reinforced foundation (0.82 mm) is decreased by 36.92% compared to the conventional geogrid-reinforced foundation (1.30 mm). Additionally, the top surface subsidence of the H–V geogrid-reinforced foundation (8.11 mm) is decreased by 13.63% compared to the conventional geogrid-reinforced foundation (9.39 mm). Figure 7 also shows that the deformation of soil in the H–V geogrid-reinforced foundation is significantly reduced compared to those in the conventional geogrid-reinforced foundation. The vertical elements of H–V geogrid play a limiting and blocking role on soil, which creates friction and an interlocking effect between the sand and the horizontal elements, making the foundation more stable. When *u* is 1B, the maximum surface uplift of the H–V geogrid-reinforced foundation and conventional geogrid-reinforced foundation is 0.39 mm and 0.71 mm, respectively. Additionally, the top surface subsidence of the H–V geogrid-reinforced foundation and conventional geogrid-reinforced foundation is 5.81 mm and 5.16 mm, respectively. It can be seen that the reinforcing effect of the H–V geogrid at this time is equivalent to that of the conventional geogrid. When the *u* is too large, the unreinforced zone directly below the footing becomes thicker. As a result, the surcharge applied by the footing is concentrated on the unreinforced soil mass above the H–V geogrid, where local failure tends to occur. Therefore, when *u* is 1B, it is recommended to use conventional geogrid to improve the foundation performance. As H–V geogrid consumes more materials, it is recommended to use conventional geogrid to improve the foundation performance when *u* is 1B. Additionally, it can be observed from Figure 10 that the surface settlement profile of the H–V geogrid-reinforced foundation is less variable at *u* = 1B than at *u* = 0.33B. Moreover, according to the strain–influence diagram proposed by Schmertmann et al. [38], the maximum vertical strain induced by a foundation occurs at 0.5B. Therefore, increasing the depth of the top H–V geogrid layer to a specific value (within the range of 0.33B to 1B) can reduce uneven settlement of the ground surface.

### 3.3. Effect of the Length of Reinforcement

**Figure 11:**
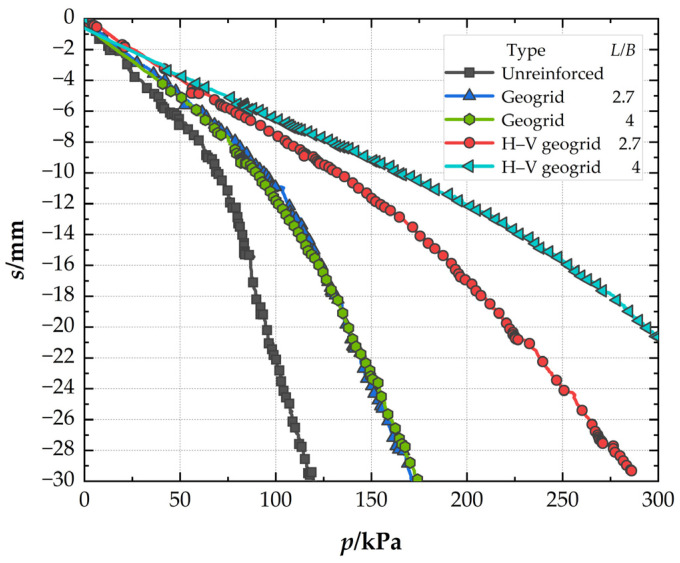
Pressure–settlement curves for conventional geogrids and H–V geogrids with different lengths of the reinforcement.

Figure 11 presents the *P*–*s* curves to investigate the effect of the length of conventional geogrid and H–V geogrid. At the settlement of 0.03B (*s* = 4.5 mm)*,* when the length of the reinforcement is 2.7B, the *BCR* of H–V geogrid-reinforced foundation (1.78) is 21.92% higher than that of conventional geogrid-reinforced foundation (1.46). When the reinforcement length is 4B, the *BCRs* of the H–V geogrid-reinforced foundation and conventional geogrid-reinforced foundation are 2.20 and 1.34, respectively. The bearing capacity improved by the H–V geogrid is also almost 1.7 times greater than the conventional geogrid. In comparison, the *BCR* of the conventional geogrid-reinforced foundation decreases (8.22%). It can be seen that increasing the length of the reinforcement to 4B helps to increase the bearing capacity of the foundation of the H–V geogrid-reinforced foundation. At the same time, it has little effect on the conventional geogrid-reinforced foundation. At the pressure of 119 kPa, when the reinforcement length is 2.7B, the *SRF* of H–V geogrid-reinforced foundation (29.6%) is 40.2% lower than that of conventional geogrid-reinforced foundation (49.5%). However, when the reinforcement length increases to 4B, the *SRF* of the H–V geogrid-reinforced foundation decreases to 24.89%, while that of conventional geogrid-reinforced foundation rises to 54.42%. Therefore, when the reinforcement length increases, the settlement of the H–V geogrid-reinforced foundation can be improved, but the conventional geogrid-reinforced foundation still needs to be improved. The optimal length of the H–V geogrid is 4B.

### 3.4. Effect of the Vertical Geogrid Height

**Figure 12:**
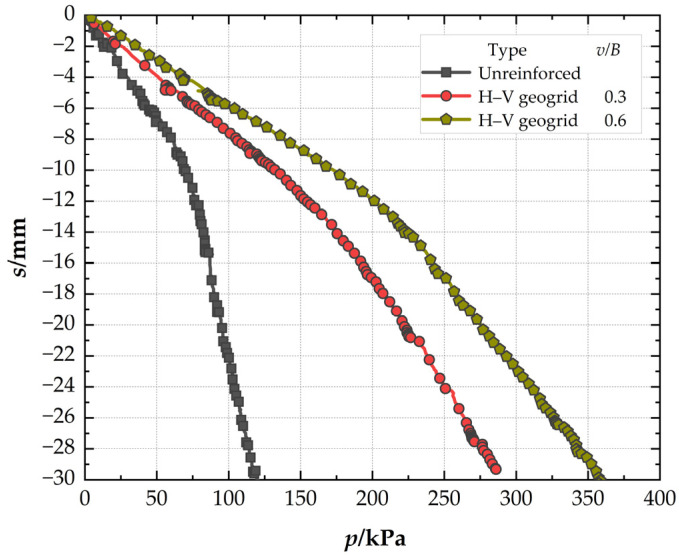
Pressure–settlement curves for H–V geogrids with different vertical geogrid heights.

Figure 12 illustrates the effect of the vertical geogrid height. At the settlement of 0.03B (*s* = 4.5 mm), the *BCR* is 2.46 for H–V reinforced foundation when *v* is 0.6B, which is 38.98% higher than when *v* is 0.3B (1.77). It can be seen that with an increase in the height of the vertical element of the H–V geogrid, the bearing capacity of the H–V reinforced foundation will increase. At the pressure of 119 kPa, the *SRF* is 22.62% for H–V reinforced foundation when *v* is 0.6B, which is 24.84% lower than when *v* is 0.3B (29.7%). So, the settlement of the H–V reinforced foundation is reduced with an increase in the vertical geogrid height (*v*). The optimal height of the H–V geogrid is 0.6B.

### 3.5. Effect of Number of H–V Geogrid Layers

**Figure 13:**
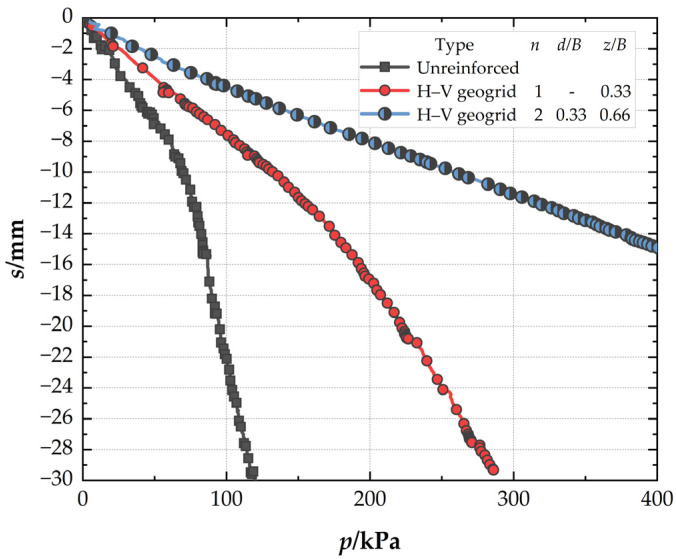
Pressure–settlement curves for H–V geogrids with different numbers of layers.

Figure 13 presents the *P*–*s* curves to investigate the effect of the number of H–V geogrid layers. As shown in Figure 13, when the settlement is 0.03B (*s* = 4.5 mm), the *BCR* of the single-layer H–V geogrid-reinforced foundation is 1.78. Meanwhile, the *BCR* of a double-layer H–V geogrid-reinforced foundation is 3.12, which is higher by 75.28% than the single-layer case. This indicates that the double-layer H–V geogrids show a better reinforcement mechanism than the single-layer H–V geogrid. When the pressure is 119 kPa, the *SRF* of single-layer H–V geogrid is 29.6%, and that of double-layer H–V geogrids is 17.5%. It can be seen that the foundation settlement can be significantly reduced by increasing the number of H–V geogrid layers. Therefore, the performance of the foundation can be improved by increasing the number of H–V geogrid layers. The optimal number of reinforced layers for H–V geogrids is two.

### 3.6. Failure Mode and Possible Mechanism

**Figure 14:**
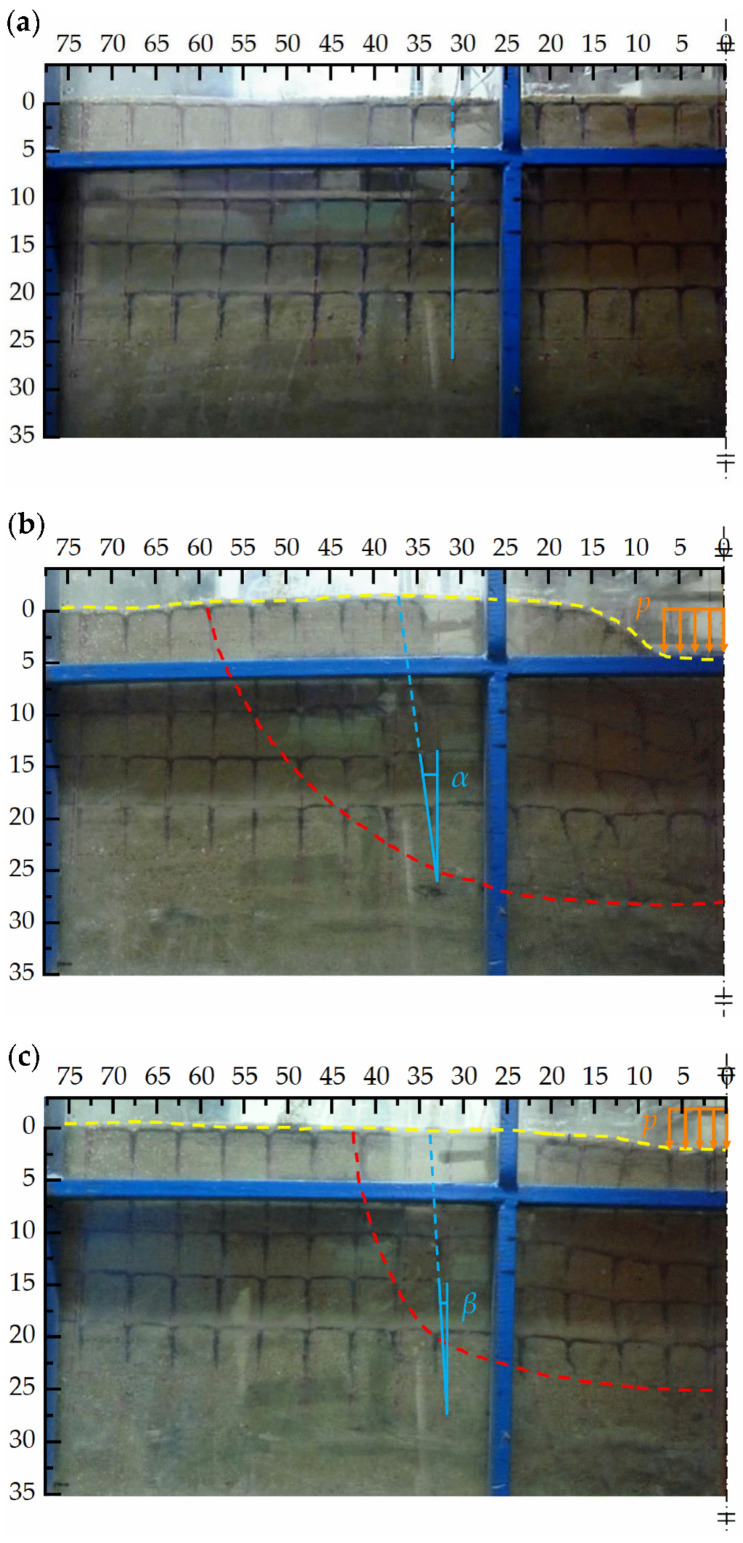
The failure of the conventional geogrid and H–V geogrid-reinforced foundation: (**a**) Unreinforced foundation (before loading); (**b**) Conventional geogrid-reinforced foundation (after loading); (**c**) H–V geogrid-reinforced foundation (after loading).

The failures of the conventional geogrid and H–V geogrid-reinforced foundations are investigated through the photos shown in Figure 14. The changing of the group of coal ash lines (black box in sand) can reveal the critical slip surface of soil applicable in reinforced foundation obviously (see the red dash lines marked in Figure 14b,c). The yellow dashed line also shows the surface deformation. It can be found that the range of slip surfaces of conventional geogrid-reinforced foundations is more significant than that of H–V geogrid-reinforced foundations. For example, at the position 27.5 cm from the center of the footing, the angle of *α* and *β* are 25° and 5°, respectively, which indicates that the stiffness and shear strength of the H–V geogrid-reinforced foundation is greater than that of a conventional geogrid-reinforced foundation. The reinforcement mechanism of the H–V geogrid works like a large mattress that spreads the applied surcharge over a wider area instead of solely at the area of contact with the footing. This leads to a composite slab that has increased flexural stiffness and load bearing capacity, resulting in the improved overall performance of the structure. 

**Figure 15:**
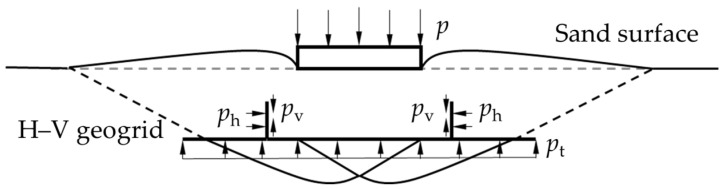
Schematic diagram of H–V geogrid mechanisms.

The difference between the H–V geogrid-reinforced foundation and the conventional one is probably due to several factors. In the case of a conventional geogrid-reinforced foundation, the frictional and interlock mechanism increases the soil’s shear strength, as investigated by many researchers [39,40,41]. However, as illustrated in Figure 15, the H–V geogrid offers confinement in three different ways. First is the friction and interlocking between the sand and the horizontal elements (*p*_h_). Second is the friction and interlocking between the sand and the vertical elements (*p*_v_). Thirdly, the H–V geogrid restrains the soil from moving up or down outside of the loading area as a mattress (*p*_t_). Therefore, due to the height of the vertical elements and their confinement, the H–V geogrid increased the stiffness of the reinforced base, redistributed the stress into a wider area, kept the sand from being displaced under the applied load, and thereby increased the shear strength of the composite system.

## 4. Conclusions

The study firstly developed a new H–V geogrid and then investigated the potential benefits of using H–V geogrids to increase the bearing capacity and reduce settlement under a strip footing through laboratory model tests. The results showed that the bearing capacity improved by the H–V geogrid is almost 1.7 times greater than the conventional geogrid. The maximum top subsidence of H–V geogrid-reinforced foundation decreased by 13.63% compared to that of conventional geogrid-reinforced foundation. When the length is 2.7 and 4 times the footing width, the bearing capacity improved by the H–V geogrid is almost 1.7 times greater than the conventional geogrid. At the given settlement of 0.03B, the bearing capacity is 38.98% higher, with the height of the vertical geogrid increasing from 0.3 times the width of the footing to 0.6. Under the same settlement, the bearing capacity ratio of two H–V geogrid-reinforced foundation layers is 75.28% higher than that of one layer. Therefore, the optimal depth of the top layer, length, and height of the vertical elements for H–V geogrids-reinforced foundations were around 1B, 4B, and 0.6B, respectively, and the optimal number of reinforced layers was two. The results also demonstrate that vertical elements of H–V geogrids provided strong passive resistance and effectively prevented the displacement of sand, thus contributing to the interfacial shear resistance of the foundation. Furthermore, the load was distributed over a broader area in the H–V geogrid-reinforced foundation compared to the conventional geogrid-reinforced foundation. However, it should be noted that these experimental results were obtained under specific testing conditions with a particular type of H–V geogrid, sand, and footing size. Therefore, it is imperative to thoroughly compare soil granules (considering factors such as size, density, and gradation) and geogrid mesh and spacing in future studies before implementing H–V geogrids in practical engineering applications.

## Figures and Tables

**Table 1:** Physical properties of sand.

The Angle of Internal Friction *φ*/°	Moisture Content *ω*/%	Relative Density of Particle *d*_s_	Unit Weight *γ*/kN/m^3^
35.2	0.15	2.63	16.74

**Table 2:** Summary of mechanical properties of the geogrid.

Polymer Type	Aperture Size	Thickness	Peak Tensile Strength	Strain at Break
HDPE	45 mm × 40 mm	2 mm	12.5 kN/m	2.5%

**Table 3:** Summary of experimental cases under study (B = 0.15 m).

No.	Type	*N*	*u*/B	*z*/B	*L*/B	*l*/B	*v*/B	*d*/B
A-1	Unreinforced							
B-1	Conventional geogrid	1	0.33	0.33	2.7			
B-2			1	1	2.7			
B-3			0.33	0.66	4			
C-1	H–V geogrid	1	0.33	0.33	2.7	1.5	0.3	
C-2			1	1	2.7	1.5	0.3	
C-3			0.33	0.33	4	1.5	0.3	
C-4			0.33	0.33	2.7	1.5	0.6	
C-5		2	0.33	0.66	2.7	1.5	0.3	0.33

## Data Availability

The data presented in this study are available on request from the corresponding author.
